# ﻿A new species of *Ampithoe* (Amphipoda, Ampithoidae) from Korea, with a redescription of *A.tarasovi*

**DOI:** 10.3897/zookeys.1079.73443

**Published:** 2021-12-22

**Authors:** Myung‐Hwa Shin, Charles Oliver Coleman

**Affiliations:** 1 National Marine Biodiversity Institution of Korea, Seochun, 325–902, Republic of Korea National Marine Biodiversity Institution of Korea Seochun Republic of Korea; 2 Museum für Naturkunde Berlin, Invalidenstraße 43, 10115 Berlin, Germany Museum für Naturkunde Berlin Berlin Germany

**Keywords:** *Ampithoechangbaensis* sp. nov., Crustacea, new species, Russia, taxonomy

## Abstract

An ampithoid species, previously known as *A.tarasovi* in Korea, is assigned as a new species, *A.changbaensis***sp. nov.** based on the reexamination of the holotype of *A.tarasovi.* The new species shows different morphological characteristics compared to *A.tarasovi*, especially in male gnathopods 1 and 2. The type material of *A.tarasovi* is redescribed and illustrated here and a key to the Korean species of *Ampithoe* is provided.

## ﻿Introduction

The genus *Ampithoe* Leach, 1814 is a herbivorous amphipod group associated with algae and seagrasses in coastal regions worldwide (Myers and Lowry 2003; Shin et al. 2015; Peart and Ahyong 2016). *Ampithoe* is the most speciose genus of the family Ampithoidae and includes more than 70 species worldwide (Horton et al. 2021).

To date, eight species of the genus *Ampithoe* have been reported from Korea: *A.akuolaka* J.L. Barnard, 1970; *A.brevipalma* Kim & Kim, 1988; *A.lacertosa* Bate, 1858; *A.ramondi* Audouin, 1826; *A.shimizuensis* Stephensen, 1944; *A.tarasovi* Bulycheva, 1952; *A.valida* Smith, 1873; and *A.youngsanensis* Kim & Kim, 1988 (Kim and Kim 1988; Shin et al. 2010; Kim 2011; Jung and Yoon 2014; Peart and Ahyong 2016). Among them, *A.lacertosa*, collected in Korea, was described by Kim and Kim (1987, 1988). After the study, the material of the Korean specimens of *A.lacertosa* was stored in the collections of the Seoul National University. Later, the deposited Korean material identified as *A.lacertosa* was reexamined in a taxonomic study of Shin et al. in 2010, and in this material a second species identified as *A.tarasovi* was found. These two species, however, were identified based on the original descriptions and other published records only. However, the type specimens of these two species were not examined by Shin et al. (2010).

For a precise identification of species, type specimens and detailed original descriptions are essential for taxonomy and a flawless identification. If type material is lost and the original texts and illustrations are short and poor in quality, it may lead to misidentifications of species.

In this study, the holotype of *A.tarasovi* collected in Russia was reexamined. Through the examination, the Korean material of *A.tarasovi* was identified as a distinct species having morphological characteristics differing from the type material of *A.tarasovi*. The examined specimens (previously known as *A.tarasovi* in Korea) are assigned as a new species, *A.changbaensis* sp. nov., which is described based on the specimens previously misidentified as *A.tarasovi* by Shin et al. (2010). Moreover, the type material of *A.tarasovi* is redescribed and illustrated.

## ﻿Materials and methods

To designate the type material of the new species, the specimens which have been deposited at the Laboratory of Systematics and Molecular Evolution in the Seoul National University were used. Other material was collected in Korea among algae in tide pools, in the intertidal zone, and in shallow water at low tide. The holotype of *Ampithoetarasovi* was loaned from the Moscow Museum, Russia, and examined at the crustacean department of Museum für Naturkunde Berlin, Germany.

The specimens were analyzed and pencil drawn under a dissection microscope (Leica M250C), and appendages and mouthparts were drawn under a Leica DMLB; both microscopes were equipped with a camera lucida. The line drawings were made using the technique described by Coleman (2003, 2009). Body length was measured along the midbody line from the tip of the rostrum to the posterior end of urosomite 3. All examined material is currently being deposited at the Marine Arthropod Depository Bank of Korea (MADBK). The descriptions were produced from a DELTA (Dallwitz 2005) database to the ampithoid genera and species (initially compiled by our colleague Dr Jim Lowry).

## ﻿Results

### ﻿Systematics

**Ampithoidae Boeck, 1871**

#### *Ampithoe* Leach, 1814

##### ﻿ Ampithoe changbaensis sp. nov.

Taxon classificationAnimalia

9C36F952-6288-5CA4-A74C-6C396B2703C2

http://zoobank.org/14B275AB-4C6A-4390-BA22-E844D6DC044E

Figures 1 , 2 , 3

Ampithoe lacertosa : Kim and Kim 1987: 3, fig. 2. Kim and Kim 1988: 109, fig. 2A [not Ampithoelacertosa Bate, 1858].
Ampithoe tarasovi : Shin et al. 2010: 300, figs 4–6 [not Ampithoetarasovi Bulycheva, 1952].

###### Type locality.

Hamo beach, Jeju-do, South Korea.

###### Type material.

***Holotype*.** Male, 17.6 mm (MABIK CR00248547), Hamo beach, Daejeong-eup, Seogwipo-si, Jejo-do, Korea (33°12'37.01"N, 126°15'44.34"E), 30 May 2007, coll. Shin and Hong.

***Paratype*.** Female, 22.3 mm (MABIK CR00248548); male and female, 16–21 mm (MABIK CR00248549); 2 males and 1 female, 17–20 mm (MABIK CR00248550), same data as the holotype.

###### Additional material examined.

3 males, Hamo beach (MABIK CR00248551), Daejeong-eup, Seogwipo-si, Jeju-do, Korea (33°12'37.01"N, 126°15'44.34"E), 30 May 2007; 3 males and female (MABIK CR00248552), Gujwa-eup, Jeju-si, Jeju-do, Korea (33°32'2.58"N, 126°50'27.25"E), 15 Mar. 2017; male and 3 females (MABIK CR00248553), Deajin port, Hyeonnae-myeon, Goseong-gun, Gangwon-do, Korea (38°29'55.42"N, 128°25'35.53"E), 21 Jun. 2019.

###### Etymology.

The new species is named in honor to Prof. Chang Bae Kim, an early amphipodologist of Korea, who collected and described the species firstly from Jeju, Korea in 1987.

###### Description.

**Figure 1.**
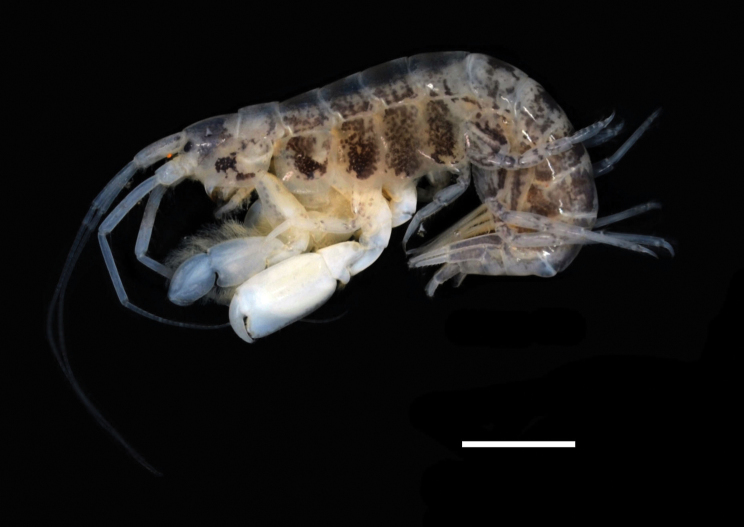
Photograph of the *Ampithoechangbaensis* sp. nov., habitus of the male paratype. Scale bar: 5 mm. Photograph by Jin-Ho Park.

**Figure 2.**
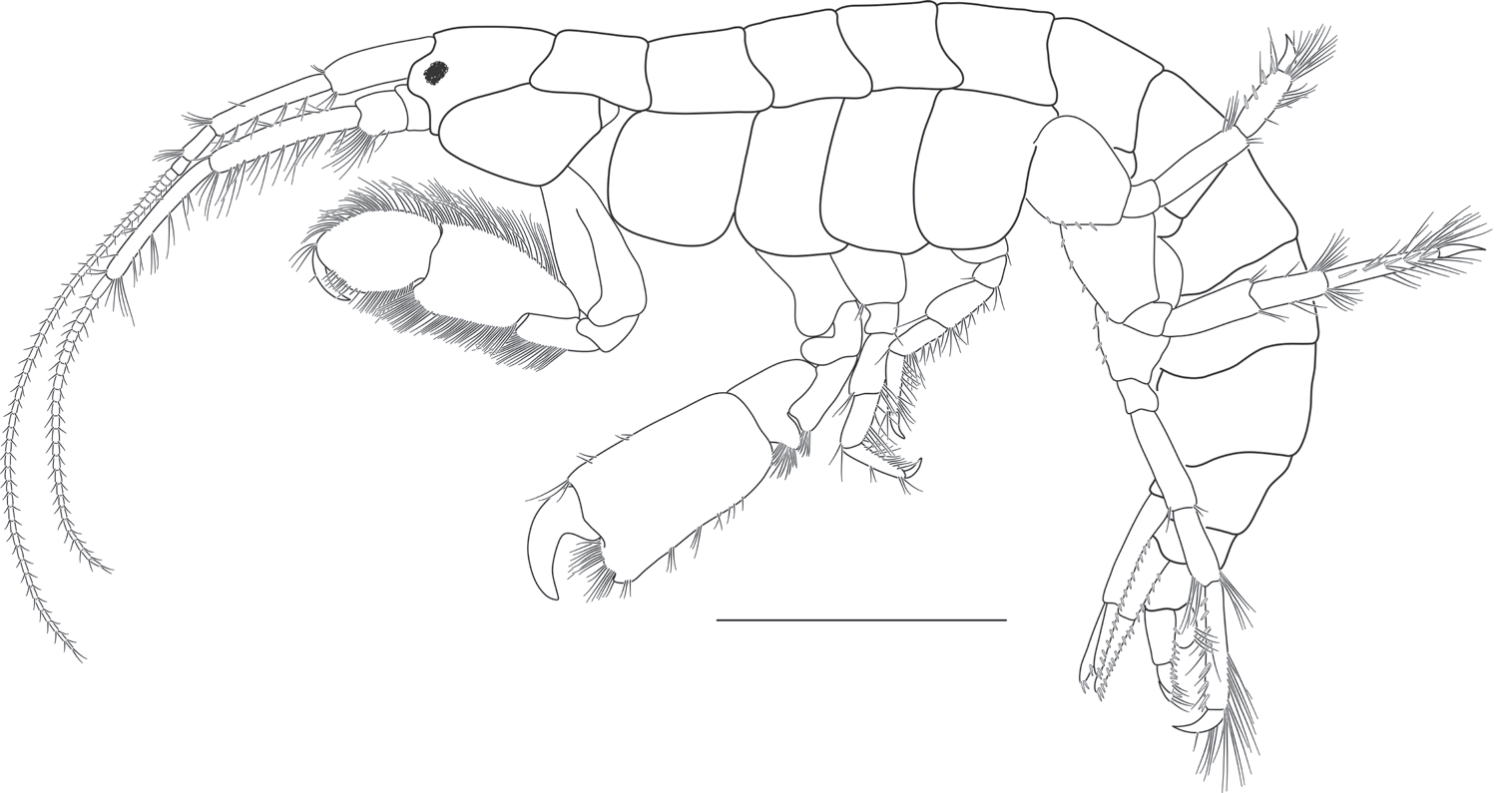
*Ampithoechangbaensis* sp. nov., male holotype. Habitus. Scale bar: 5 mm.

Based on holotype male, 17.6 mm. Body (Figs 1, 2) heavily covered with dark pigmentation spots creating bands on head, coxae, pereon, and pleon.

Head. Antenna 1 longer than antenna 2; peduncular article 1 subequal in length to article 2; article 2 longer than article 3 (2.3 times article 3); article 3 shorter than article 1 (0.5 times article 1).

Antenna 2 slender, similar to antenna 1; peduncular article 4 subequal in length to article 5; flagellum longer than peduncular article 5.

Upper lip with midlateral notch on margins.

Mandible molar well developed, triturating; palp apically setose, 3-articulate; mandibular palp article 1 shorter than article 2 (0.5 times article 2); article 2 shorter than article 3 (0.7 times article 3); article 3 longer than article 1 (3 times article 1).

Lower lip outer plates forming a medial excavation, lateral lobe slightly longer than medial lobe; mandibular lobe curved laterally, subacute apically.

Maxilla 1 inner plate with 1 slender seta; palp well developed, with apical robust setae.

Maxilla 2 inner plate narrower than outer plate, with oblique setal row.

Maxilliped outer plate with developed row of large robust setae along medial margin.

**Figure 3.**
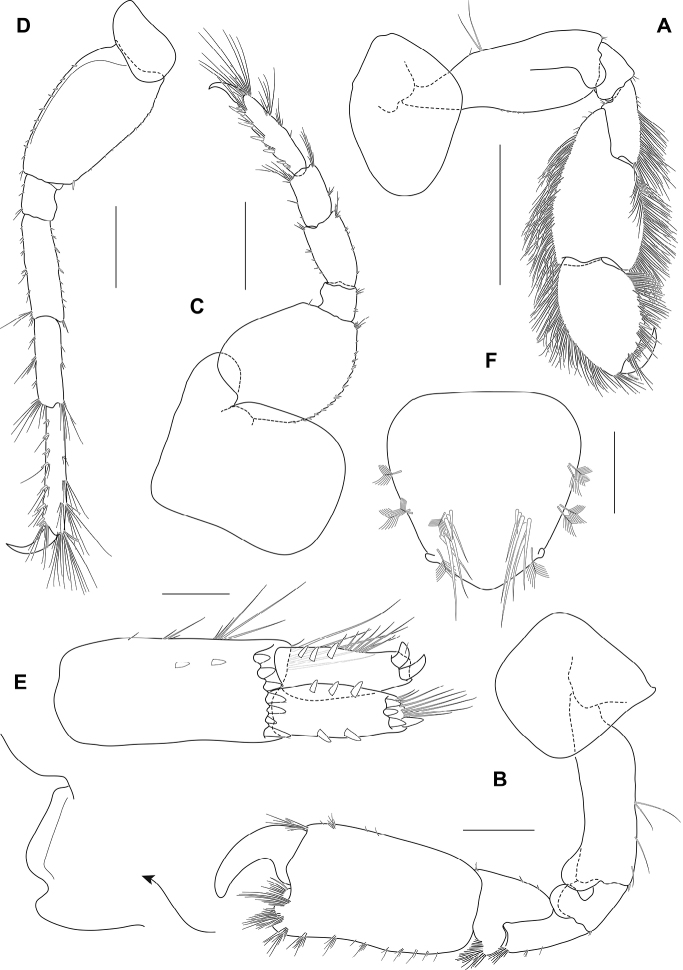
*Ampithoechangbaensis* sp. nov., male holotype **A** gnathopod 1 **B** gnathopod 2 **C** pereopod 5 **D** pereopod 7 **E** uropod 3 **F** telson. Scale bars: 2 mm (**A–D**); 0.25 mm (**E, F**).

Pereon. Gnathopod 1 (Fig. 3A) sexually dimorphic, smaller than gnathopod 2, carpus and propodus with numerous plumose setae on both anterior and posterior margins; coxa broader than deep, anterior margin slightly convex, anteroventral corner produced, rounded; basis longer than coxa, expanded anterodistally, anterodistal lobe large and subrounded; ischium anterior margin with small subrounded lobe; merus posterodistal corner subacute, produced; carpus about 2 times as long as broad, longer than propodus (1.3 times propodus), with posterodistal lobe slightly overlapping propodus, posterior margin slightly convex; propodus broad, 1.4 times as wide as long, subovoid; palm acute, convex, defining corner rounded with 1 robust seta; dactylus subequal in length to palm.

Gnathopod 2 (Fig. 3B) sexually dimorphic; basis longer than coxa, anterodistal lobe large and rounded, not reaching beyond ischium; ischium with anterior rounded lobe; carpus much shorter than propodus (0.4 times propodus), subtriangular; propodus narrow, 1.8 times as long as wide, subrectangular; palm transverse, with a sloped quadrate midmedial hump and an apically rounded tooth on posterodistal corner; dactylus slightly overreaching palm, curved, robust, apically blunt.

Pereopod 3 basis narrow; merus narrow; carpus about twice as long as broad.

Pereopod 4 basis similar to pereopod 3.

Pereopod 5 (Fig. 3C) basis subovoid, without posterodistal lobe; merus subrectangular.

Pereopod 6 basis posterior margin rounded proximally, straight distally, with marginal robust setae; merus subrectangular.

Pereopod 7 (Fig. 3D) similar to pereopod 6; basis with marginal robust setae.

Pleon. Epimera 1–3 with lateral ridges; epimera 2 and 3 subrounded posterodistally, with rounded tooth on each posteroventral angle. Epimeron 1 rounded posterodistally, with tooth on posteroventral angle; epimeron 2 ventral margin evenly curved; epimeron 3 ventral margin straight.

Uropod 1 reaching to end of uropod 2 rami; inner ramus longer than outer ramus; outer ramus slender, about 6 times as long as broad.

Uropod 2 inner ramus longer than outer ramus.

Uropod 3 (Fig. 3E) peduncle much longer than broad (2.2 times as wide as long), 1.8 times as long as rami, 2 inner marginal robust setae, marginal slender setae present, with 7 distal peduncular robust setae; rami long, about twice as long as broad; outer ramus subequal in length to inner ramus, with 2 large recurved distal robust setae, and with 3 dorsomarginal robust setae, with lateral setal fringe; inner ramus with 4 distal robust setae, with 3 and 2 lateral robust setae on both inner and outer margins, respectively.

Telson (Fig. 3F) subtriangular, apically rounded, with small apical cusps; with 3 groups of lateral plumose setae, and a pair row of setae on submedial margins.

###### Depth zone.

Littoral (1–2 m).

###### Distribution.

Jeju, East Sea of Korea, South Sea of Korea.

###### Remarks.

This species has been recognized as *A.tarasovi* in Korea since the species was described and illustrated by Shin et al. (2010). However, the type material of *A.tarasovi* described from Russia was examined here, and as a result, important morphological differences were detected between Korean material and the Russian type specimen. The characteristics differing between the Korean specimens and the type material are as follows. In male gnathopod 1, the basis is expanded anterodistally in the Korean specimens, while it is narrow and straight in the type material. The length ratios of carpus and propodus of the Korean and Russian specimens are 1.3 and 1.8, respectively. The shape of gnathopod 1 carpus is more rectangular in the Russian material than in the Korean sample. The propodus of male gnathopod 2 is longer and more rectangular in the Russian material compared to the Korean sample. The apical margin of the telson is round in the Korean specimen, while it is subacute in the Russian type material. Based on the morphological differences mentioned above, the Korean material has been assigned to a new species, *A.changbaensis* sp. nov.

*Ampithoechangbaensis* sp. nov. is similar to *A.prolata* Hughes & Peart, 2013; however, it can be distinguished from this species by the following characteristics: (1) presence of marginal setae on merus, carpus and propodus of male gnathopod 1; (2) swollen basis of male gnathopod 1; (3) subrectangular and trapezoid shape of propodus of male gnathopod 2; and (4) truncated posterior margin of carpus of female gnathopod 1.

##### ﻿ Ampithoe tarasovi

Taxon classificationAnimalia

Bulycheva, 1952

03A178DC-B3CD-595F-A3E8-7D787B3BD386

**Figure 4.**
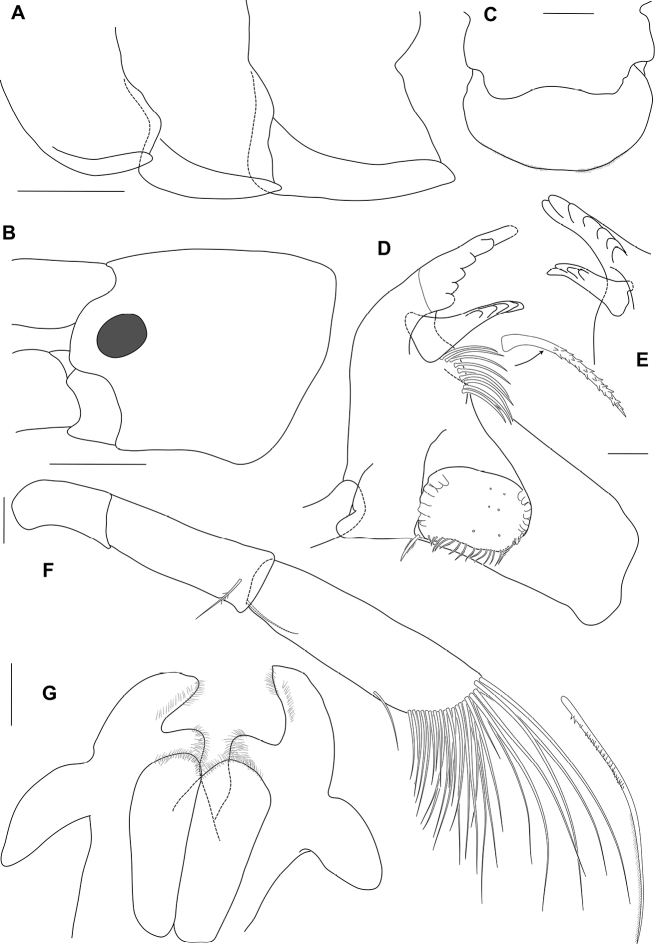
*Ampithoetarasovi* Bulycheva, male holotype **A** epimeral plates 1–3 **B** head **C** upper lip **D** left mandible **E** right mandible **F** palp of mandible **G** lower lip. Scale bars: 1 mm (**A, B**); 0.2 mm (**C, G**); 0.1 mm (**D–F**).

**Figure 5.**
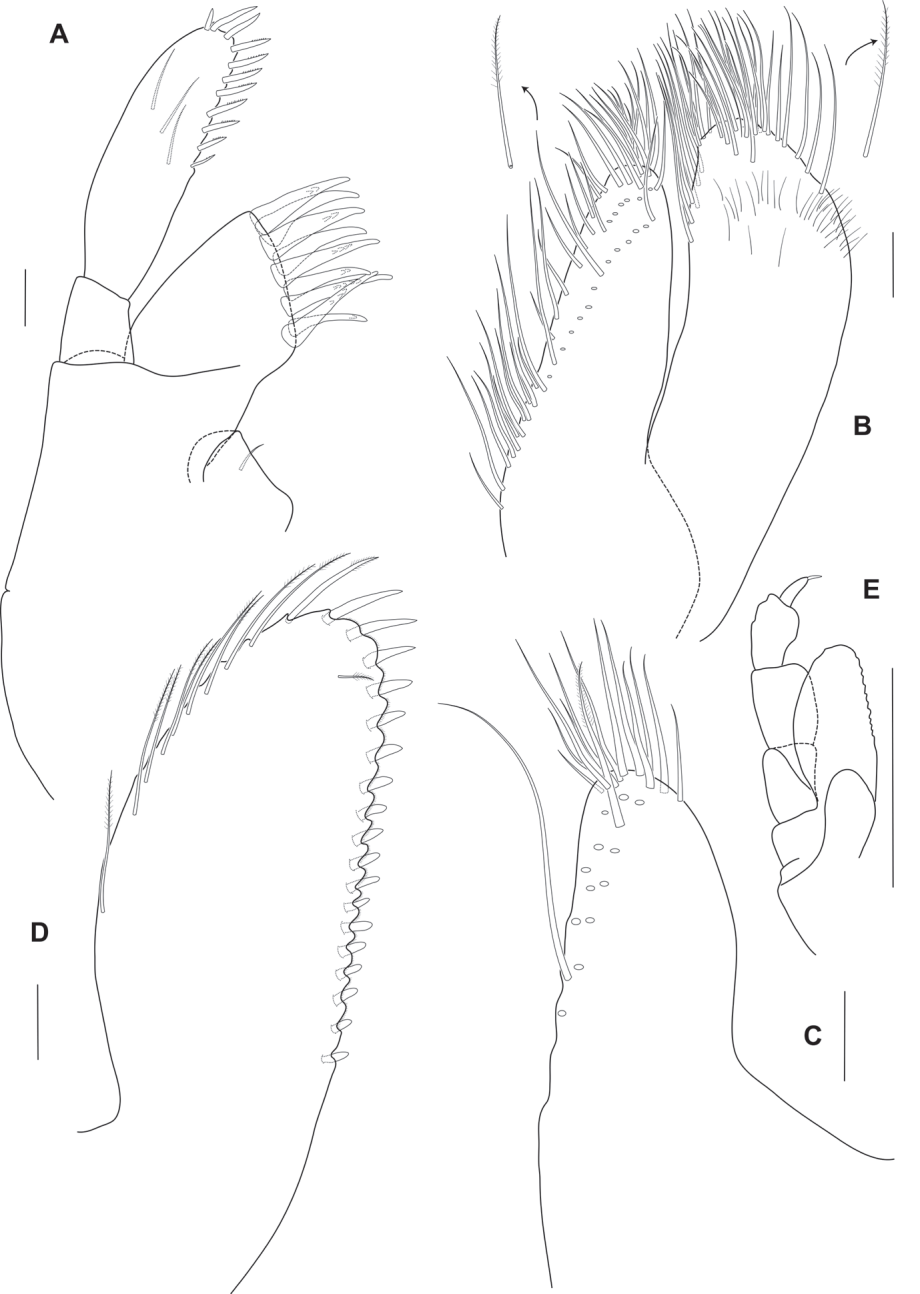
*Ampithoetarasovi* Bulycheva, male holotype **A** maxilla 1 **B** maxilla 2 **C** inner lobe of maxilliped **D** outer lobe of maxilliped **E** maxilliped. Scale bars: 0.1 mm (**A–D**); 1 mm (**E**).

**Figure 6.**
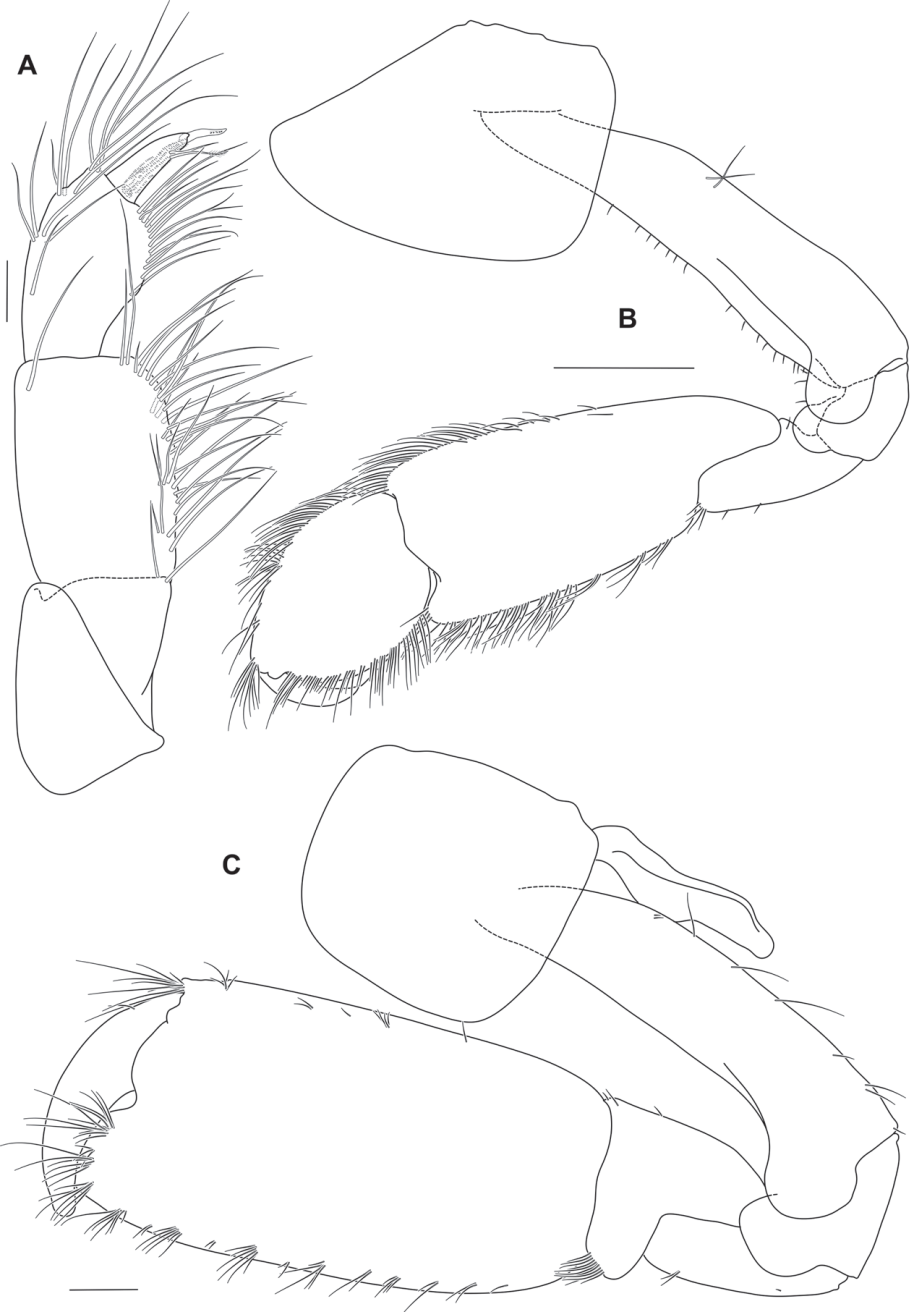
*Ampithoetarasovi* Bulycheva, male holotype **A** palp of maxilliped **B** gnathopod 1 **C** gnathopod 2. Scale bars: 0.1 mm (**A**); 1 mm (**B**); 0.5 mm (**C**).

**Figure 7.**
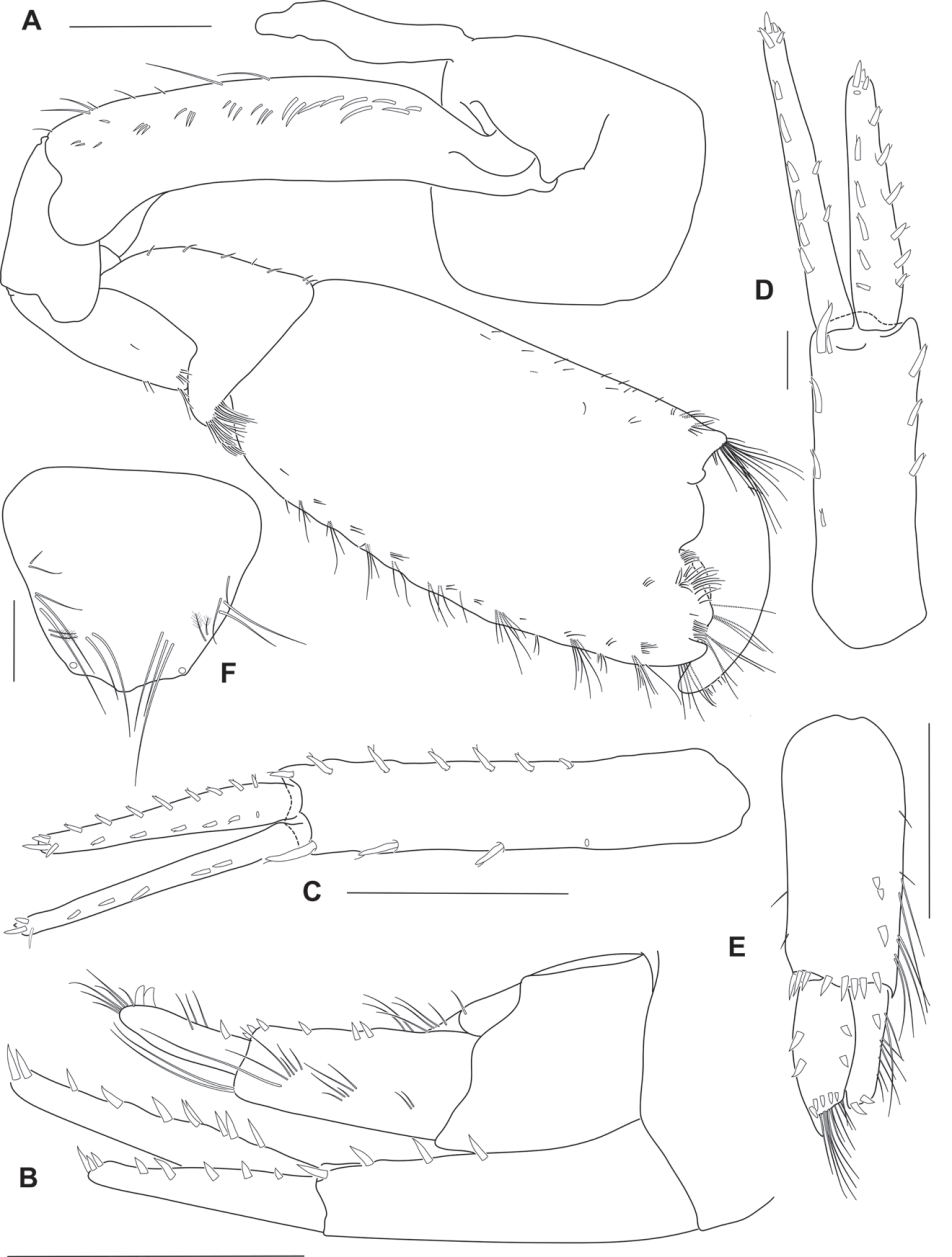
*Ampithoetarasovi* Bulycheva, male holotype **A** gnathopod 2 **B** lateral view of urosomite 3 **C** uropod 1 **D** uropod 2 **E** uropod 3 **F** telson. Scale bars: 1 mm (**A–C, E**); 0.2 mm (**D, F**).

**Figure 8.**
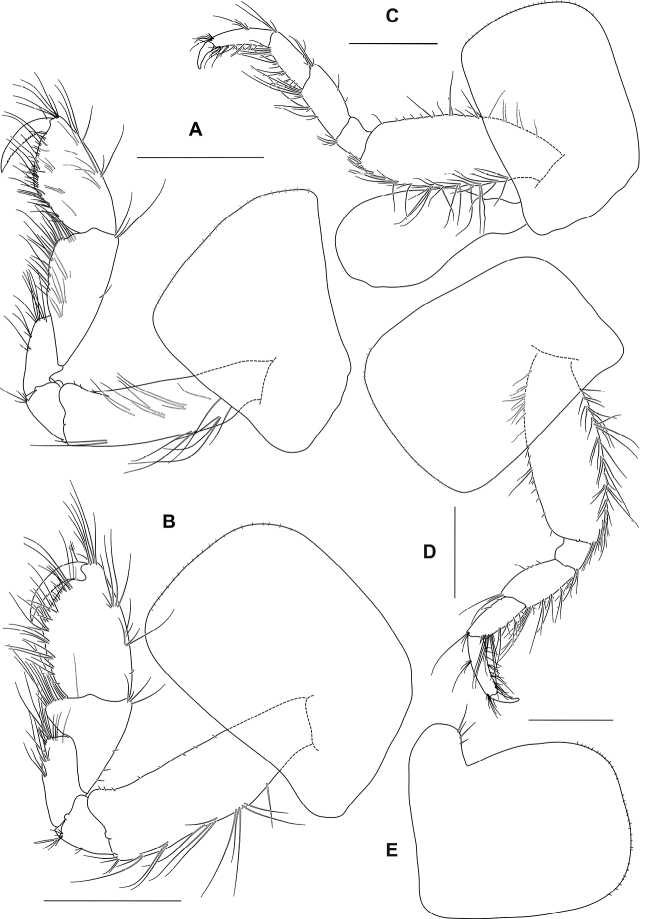
*Ampithoetarasovi* Bulycheva, female **A** gnathopod 1 **B** gnathopod 2 **C** pereopod 3 **D** pereopod 4 **E** coxa 5. Scale bars: 1 mm (**A–E**).

Figures 4 , 5 , 6 , 7 , 8

Ampithoe tarasovi Bulycheva, 1952: 246, fig. 38. Tzvetkova 1967: 190.

###### Type material.

Male, collected by Tarasov from De-Kastri, Sea of Japan, 3 Aug. 1929.

###### Description.

Based on holotype male, 14.3 mm (re-measured along the midbody line from the tip of the rostrum to the posterior end of urosomite 3), deposited at the Moscow Museum, Russia (no. 1/21349).

Head (Fig. 4B). Upper lip (Fig. 4C) with mid-lateral notch on margins.

Mandible (Fig. 4D, E) molar well developed, triturating; accessory setal row with 9 robust setae; palp apically setose, 3-articulate; mandibular palp (Fig. 4F) article 1 shorter than article 2 (0.6 times article 2); article 2 shorter than article 3 (0.7 times article 3); article 3 long (3.3 times as long as wide), longer than article 1 (2.3 times article 1).

Lower lip (Fig. 4G) outer plates forming a medial excavation, lateral lobe much longer than medial lobe; mandibular lobe curved laterally, rounded apically.

Maxilla 1 (Fig. 5A) inner plate with 1 slender seta; palp well developed, with apical robust setae.

Maxilla 2 (Fig. 5B) inner plate narrower than outer plate, with oblique setal row.

Maxilliped (Figs 5C–E, 6A) outer plate with developed row of large robust setae along medial margin.

Pereon. Gnathopod 1 (Fig. 6B) sexually dimorphic, smaller than gnathopod 2, carpus and propodus with numerous plumose setae on both anterior and posterior margins; coxa subequal to coxa 2 in length, broader than deep, anterior margin straight, anteroventral corner produced, rounded; basis longer than coxa, anterodistal lobe large and rounded; ischium anterior margin with large rounded lobe; merus posterodistal corner subquadrate; carpus about 2 times as long as broad, longer than merus, longer than propodus (1.8 times propodus), with posterodistal lobe slightly overlapping propodus, posterior margin straight; propodus broad, 1.4 times as wide as long, subovoid; palm acute, convex, defining corner rounded with 1 robust seta; dactylus subequal in length to palm.

Gnathopod 2 (Figs 6C, 7A) sexually dimorphic; basis longer than coxa, with sparse slender setae, anterodistal lobe large and rounded, not reaching beyond ischium; ischium anterior margin with subquadrate lobe; carpus much shorter than propodus (0.3 times propodus), subtriangular; propodus narrow, 2.3 times as long as wide, subrectangular; palm transverse, with a sloped quadrate mid-medial hump and an apically rounded defining tooth on posterodistal corner; dactylus slightly overreaching palm, curved, robust, apically blunt, without unguis.

Pereopod 3 (Fig. 8C) basis narrow; merus narrow; carpus about twice as long as broad.

Pereopod 4 (Fig. 8D) basis similar to pereopod 3.

Pereopod 5 (Fig. 8E) coxa simple and subrectangular. Pereopods 5–7 lost.

Pleon. Epimera 1–3 (Fig. 4A) with lateral ridges; ventral margin of epimera 2 and 3 straight, with distinct tooth on each posteroventral angle. Epimeron 1 subrounded posterodistally, with tooth on posteroventral angle; epimeron 2 subrounded posterodistally; epimeron 3 straight and sloped posterodistally.

Uropod 1 (Fig. 7B, C) reaching to end of uropod 2 rami; peduncle with 10 robust setae; inner ramus longer than outer ramus, with 5 marginal robust setae; outer ramus slender, about 6 times as long as broad, with 14 marginal robust setae.

Uropod 2 (Fig. 7D) peduncle with 8 robust setae; inner ramus longer than outer ramus, with 8 marginal robust setae; outer ramus 11 marginal robust setae.

Uropod 3 (Fig. 7E) peduncle much longer than broad (2.3 times width), 2.2 times as long as rami, with 3 marginal robust setae, marginal slender setae present, with 8 distal peduncular robust setae; rami long, about twice as long as broad; outer ramus shorter than inner ramus, with 2 large recurved distal robust setae and 1 dorsal robust setae, with lateral setal fringe; inner ramus with 6 distal robust setae, with 2 lateral robust setae both inner and outer margins, respectively.

Telson (Fig. 7F) subtriangular, apically subacute, with small apical cusps, with 4 or 5 of lateral setae on both margins, and 2 pairs of lateral plumose setae, with 2 or 3 submedial setae on both margins.

Sexual dimorphic female, 15.3 mm.

Gnathopod 1 (Fig. 8A) subequal in size to gnathopod 2; coxa about as broad as long, anterior margin concave, anteroventral corner produced and subacute; basis subequal in length to coxa, with sparse slender setae, anterodistal lobe large and rounded; carpus subequal in length to propodus (1.1 times propodus); propodus narrow, 2 times as long as wide, subtriangular; palm acute, straight, defining corner subrounded with 1 robust seta; dactylus slightly overreaching palm, inner margin crenate.

Gnathopod 2 (Fig. 8B) basis shorter than coxa, with sparse slender setae, anterodistal lobe large and rounded, not reaching beyond ischium; ischium anterior margin without distinct lobe; carpus shorter than propodus (0.7 times propodus); propodus narrow, 1.6 times as long as wide, subrectangular; palm acute, defining corner subrounded with 1 robust seta; dactylus slightly overreaching palm, tapering evenly, apically acute, inner margin crenate.

**Table 1.** Distribution of the species of *Ampithoe* in coastal regions of Korea: EC, Eastern coast; WC, Western coast; SC, Southern coast; JC, Jeju coast (distribution data cited from Kim [2011] and Jung and Yoon [2014]).

Species	Coastal region			
	EC	WC	SC	JC
*A.akuolaka*	–	–	◎	–
*A.brevipalma*	◎	–	◎	–
*A.lacertosa*	◎	◎	◎	◎
*A.ramondi*	◎	–	◎	◎
*A.shimizuensis*	◎	◎	◎	◎
*A.valida*	◎	◎	◎	◎
*A.youngsanensis*	–	–	◎	◎
*A.changbaensis* sp. nov.	◎	–	◎	◎

###### Depth zone.

Sublittoral (0–24 m).

###### Distribution.

Peter the Great Bay, Sea of Japan.

###### Remarks.

This species has the following characteristics: the apical and medial lobes of the outer lobes are separated in the lower lip; the carpus of male gnathopod 1 is about 1.8 times as long as the propodus; the palm of the male gnathopod 2 has a sloped quadrate hump and posterodistal tooth. Bulycheva (1952) noted that *A.tarasovi* is very abundant in macroalgae and reefs in Petra Velikogo Bay and in the northern Sea of Japan.

### ﻿Here we provide a key to the Korean species of *Ampithoe* and distributional information in four coastal regions of Korea in Table 1.

**Table:** 

1	In male, gnathopod 2, propodus subovoid or not large; palm acute or not	**2**
–	In male, gnathopod 2, propodus large subrectangular; palm transverse or nearly so	**5**
2	Gnathopod 2, palm extremely acute, not defined in male	***A.youngsanensis***
–	Gnathopod 2, palm not acute in male	**3**
3	Gnathopod 2, palm concave, defined with angle in male	***A.brevipalma***
–	Gnathopod 2, palm incised, with distinct lobe in male	**4**
4	Gnathopod 2, propodus with pointed thumb-like lobe in male ***A.akuolaka***
–	Gnathopod 2, propodus with rounded thumb-like lobe in male ***A.ramondi***
5	Epimeron 3 without tooth on posteroventral angle	**6**
–	Epimeron 3 with subacute tooth on posteroventral angle	**7**
6	In male antenna 2, peduncular article 4 compressed and expanded	***A.shimizuensis***
–	In male antenna 2, peduncular article 4 not expanded, ordinary	***A.valida***
7	In male, gnathopod 2, carpus and propodus with dense marginal setae; palm with quadrate hump; gnathopod 1, basis expanded anterodistally in male	***A.changbaensis* sp. nov.**
–	In male, gnathopod 2, carpus and propodus without dense setae; palm without hump; gnathopod 1, basis not expanded anterodistally in male	**7**

## Supplementary Material

XML Treatment for Ampithoe changbaensis

XML Treatment for Ampithoe tarasovi
